# A phase 2, open-label, multicenter study of the long-term safety of siltuximab (an anti-interleukin-6 monoclonal antibody) in patients with multicentric Castleman disease

**DOI:** 10.18632/oncotarget.4655

**Published:** 2015-08-03

**Authors:** Frits van Rhee, Corey Casper, Peter M. Voorhees, Luis E. Fayad, Helgi van de Velde, Jessica Vermeulen, Xiang Qin, Ming Qi, Brenda Tromp, Razelle Kurzrock

**Affiliations:** ^1^ Myeloma Institute for Research and Therapy, University of Arkansas for Medical Sciences, Little Rock, AR, USA; ^2^ Fred Hutchinson Cancer Research Center, Seattle, WA, USA; ^3^ Lineberger Comprehensive Cancer Center, University of North Carolina, Chapel Hill, NC, USA; ^4^ MD Anderson Cancer Center, The University of Texas, Houston, TX, USA; ^5^ Janssen Research & Development LLC, Beerse, Belgium; ^6^ Janssen Research & Development LLC, Leiden, Netherlands; ^7^ Janssen Research & Development LLC, Spring House, PA, USA

**Keywords:** multi-centric Castleman's disease, interleukin-6, siltuximab, clinical trial

## Abstract

**Background:**

Multicentric Castleman disease (MCD) is a rare, systemic lymphoproliferative disorder driven by interleukin (IL)-6 overproduction. Siltuximab, an anti-IL-6 monoclonal antibody, has demonstrated durable tumor and symptomatic responses in a multinational, randomized, placebo-controlled study of MCD.

**Methods:**

This preplanned safety analysis was conducted to evaluate the long-term safety of siltuximab treatment among 19 patients with MCD who had stable disease or better and were enrolled in a phase-1 study and subsequent ongoing, open-label, phase-2 extension study. Dosing was 11 mg/kg administered intravenously every 3 weeks, per protocol, or every 6 weeks at the investigator's discretion. Safety monitoring focused on potential risks associated with the anti-IL-6 mechanism of action. Investigator-assessed disease control status was also documented.

**Results:**

Median treatment duration for the 19 patients was 5.1 (range 3.4, 7.2) years, with 14 (74%) patients treated for >4 years. Grade-≥3 adverse events (AEs) reported in >1 patient included hypertension (*n* = 3) and nausea, cellulitis, and fatigue (*n* = 2 each). Grade-≥3 AEs at least possibly attributed to siltuximab were leukopenia, lymphopenia, and a serious AE of polycythemia (*n* = 1 each). Hypertriglyceridemia and hypercholesterolemia (total cholesterol) were reported in 8 and 9 patients, respectively. No disease relapses were observed, and 8 of 19 patients were able to switch to an every-6-week dosing schedule.

**Conclusions:**

All MCD patients in this extension study have received siltuximab for a prolonged duration (up to 7 years) without evidence of cumulative toxicity or treatment discontinuations and with few serious infections. All patients are alive, demonstrate sustained disease control, and continue to receive siltuximab.

## INTRODUCTION

Multicentric Castleman disease (MCD) is a rare lymphoproliferative disease driven by dysregulation of the cytokine interleukin (IL)-6 [1]. At the time of performance of the current study, no standard of care was established for MCD [2]. Systemic manifestations of MCD include fever, fatigue, night sweats, wasting, weight loss, and loss of appetite [3]. While clinical manifestations vary, enlargement of multiple lymph nodes and laboratory abnormalities (eg, anemia, hypoalbuminemia, and elevated acute-phase proteins) are common [1, 4]. Treatment with siltuximab, an anti-IL-6 monoclonal antibody with high affinity for human IL-6, has demonstrated efficacy in patients with Castleman disease treated in a phase 1 clinical trial [5, 6]. More recently, results from a randomized, double-blind, placebo-controlled study confirmed that siltuximab treatment can yield durable tumor and symptomatic responses, resolution of anemia, and improvement in inflammatory disease parameters in patients with MCD [7] and led to the recent approvals from the Unites States (US) Food and Drug Administration [8] and European Commission [9] of siltuximab for the treatment of patients with MCD who are human immunodeficiency virus (HIV) negative and human herpesvirus-8 (HHV-8) negative.

In phase 1 evaluations, the siltuximab safety profile was consistent across all dose levels tested. Thus, a recommended dose of 11 mg/kg was determined based on efficacy and adequate suppression of C-reactive protein (CRP) at that dose level. In the aforementioned randomized study [7], the incidences of grade-≥3 adverse events (AEs) and serious adverse events (SAEs) were similar between siltuximab and placebo. The most commonly reported grade-≥3 AEs included fatigue (9%) and night sweats (8%); infusion reactions were relatively infrequent (8%) and generally of low grade (1 anaphylactic reaction led to siltuximab discontinuation) [7]. This safety profile, however, relates to median treatment durations of 8.5 (range 0.03, 60.5) months (phase 1) [5] and 12.3 (range 0.03, 33.9) months (randomized study) [7]. Thus, this long-term extension study provides a unique opportunity to characterize the long-term safety profile of treatment with siltuximab in patients with MCD.

## RESULTS

### Baseline characteristics and disposition

Between April 2011 and January 2013, 19 of the 37 patients with MCD from the previous phase 1 siltuximab study [5] were enrolled in the study extension and continued to receive siltuximab at 4 US sites. When these patients initially commenced siltuximab treatment in the phase 1 study, the median age was 44 (range 18, 76) years and the median disease duration was 4.8 months (37% of patients were newly diagnosed). Among the 12 patients with prior systemic therapy, rituximab (*n* = 8) and corticosteroids (*n* = 6) were the most commonly used (Table 1). Of these 19 patients, 10 (53%) had hyaline vascular and 9 (47%) had plasmacytic histological subtype. Five patients had a body mass index (BMI) >40 when entering the extension study, and an additional 3 had a BMI >30.

**Table 1:** Patient demographic and disease characteristics at start of initial siltuximab treatment

	Extension study patients (*N* = 19)
Male	12 (63)
Race	
Caucasian	16 (84)
Black	1 (5)
Asian	2 (11)
Age, years	44 [18, 76]
Weight, kg	83.5 [55.4, 169.8]
Disease duration, months	4.8 [1.3, 93.2]
Newly diagnosed^a^	7 (37)
Karnofsky performance status score	80 [60, 100]
Histology	
Hyaline vascular	10 (53)
Plasmacytic	9 (47)
Prior therapy	
Cancer-related surgery	4 (21)
Systemic therapy	12 (63)
Rituximab	8 (42)
Corticosteroid	6 (32)
Thalidomide	2 (11)
Cyclophosphamide	1 (5)

Data presented as *n* (%) or median [range].

a Patients who had not received any prior systemic therapy.

At the time of enrollment in the extension study, the 19 participating patients had received a median of 58 (range 29, 97) doses of siltuximab over a median of 3.3 (range 1.6, 5.0) years while in the phase 1 study. From the start of the phase 1 study to the data cutoff for the current interim analysis (January 2013), these patients received a median of 81 (range 49, 129) doses of siltuximab over a median of 5.1 (range 3.4, 7.2) years, with 14 (74%) of patients treated for longer than 4 years. At the time of the analysis, 11 patients received treatment at q3wks dosing and 8 patients received treatment at q6wks dosing (for 1 patient, this meant a continuation of the dosing interval within the phase 1 study). Among these 8 patients, the median treatment duration on the extended (q6wks) dosing interval was 11 months (range 5.8 months to 3.8 years). Of note, 1 additional patient started in this study with 4 cycles q6wks dosing and continued with q3wks dosing. All 19 patients are alive and continue to receive siltuximab treatment.

### Safety

**Figure 1:**
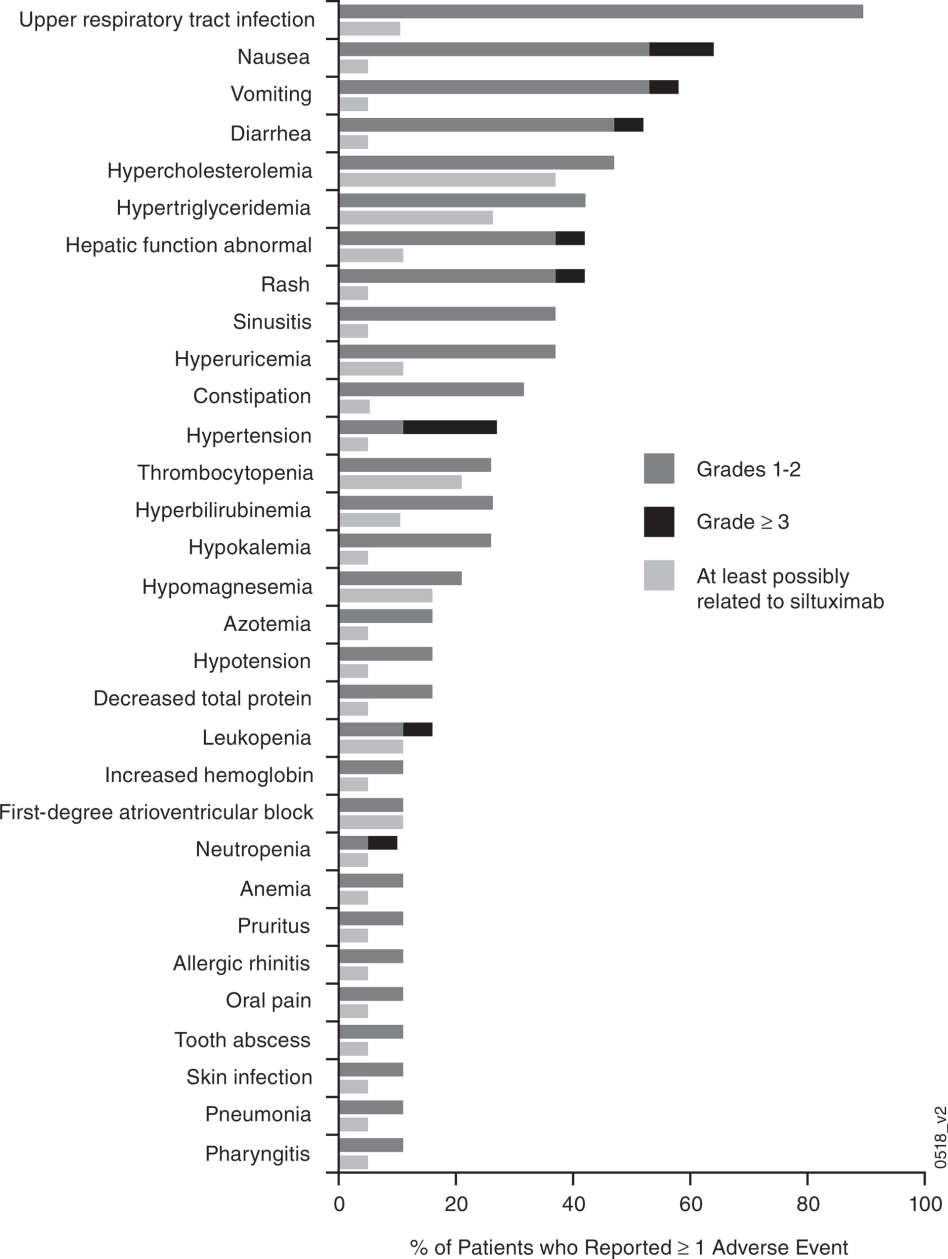
Adverse events considered at least possibly attributed to siltuximab for those events reported by ≥10% of patients across both the phase 1 and extension studies (*n* = 19) ^a^In addition, the adverse events of bone pain, herpes zoster, lymphopenia, rectal abscess, anal fistula, cardiac disorder, mitral valve incompetence, oral candidiasis, sinus bradycardia, chronic otitis media, irritable bowel syndrome, urobilinogen urine, decreased blood immunoglobulin M, and infusion-related reaction (5% each) and a serious adverse event of polycythemia (5%) were also considered at least possibly attributed to siltuximab.

Over the combined time course of the phase 1 and extension studies (median 5.1 years; range 3.4, 7.2 years), all patients reported at least 1 AE of any grade, with upper respiratory tract infection (90%); nausea (63%), vomiting (58%); diarrhea (53%); hypercholesterolemia (total cholesterol; 47%); and pain in extremities, hypertriglyceridemia, headache, rash, and hepatic function abnormal (42% each) being the most commonly reported (Figure 1). The most common AEs (>20%) reported in the extension study alone were upper respiratory tract infection (63%); diarrhea (32%); and fatigue, arthralgia, and pain in extremities (21% each). Those considered at least possibly attributed to siltuximab were reported in 6 patients during the extension study and included pneumonia, tooth abscess, constipation, increased hemoglobin, hypertriglyceridemia, hyperbilirubinemia, chronic otitis media, and upper respiratory tract infection (5% each) and grade-3 leukopenia and lymphopenia (5% each). When assessed by length of patient treatment/follow-up, the incidences of AEs for the different system organ classes were similar or lower during the treatment periods of 2–4 years and >4 years when compared with the initial 2 years of treatment (Table 2). In most patients, the highest grade of AE reported was either grade 2 (37%) or grade 3 (53%); 2 (11%) patients had a grade-4 AE. Grade-≥3 AEs were most commonly reported in the following system organ classes: gastrointestinal, 6 (32%); infections, 5 (26%); blood/lymphatic system, 4 (21%); and general disorders/administration-site, 4 (21%). Most grade-≥3 AEs were each reported in only 1 patient; exceptions included 3 (16%) patients with hypertension and 2 (11%) patients each with nausea, cellulitis, and fatigue. No patient developed an infusion-related reaction, nor did any patient develop antibodies against siltuximab during the extension study.

**Table 2:** Frequently reported^a^ adverse events by system-organ class and number of years of treatment

	Years of siltuximab treatment		
	0 < Year ≤ 2	2 < Year ≤ 4	> Year 4
Treated patients	19	19	14
Patients with adverse events	19 (100)	19 (100)	13 (93)
Infections and infestations	16 (84)	18 (95)	11 (79)
Upper respiratory tract infection	10 (53)	10 (53)	8 (57)
Nasopharyngitis	2 (11)	3 (16)	4 (29)
Urinary tract infection	5 (26)	2 (11)	1 (7)
Sinusitis	5 (26)	5 (26)	0
Gastrointestinal disorders	14 (74)	13 (68)	7 (50)
Diarrhea	8 (42)	5 (26)	5 (36)
Nausea	10 (53)	5 (26)	1 (7)
Musculoskeletal and connective tissue disorders	14 (74)	7 (37)	7 (50)
Back pain	3 (16)	1 (5)	4 (29)
Pain in extremity	7 (37)	1 (5)	4 (29)
Arthralgia	6 (32)	4 (21)	2 (14)
General disorders and administration site conditions	9 (47)	7 (39)	5 (36)
Metabolism and nutrition disorders	14 (74)	8 (42)	5 (36)
Hypertriglyceridemia	7 (37)	4 (21)	1 (7)
Hyperuricemia	4 (21)	5 (26)	1 (7)
Hypercholesterolemia	9 (47)	2 (11)	0
Skin and subcutaneous tissue disorders	9 (47)	6 (32)	5 (36)
Rash	5 (26)	5 (26)	1 (7)
Eye disorders	2 (11)	3 (16)	4 (29)
Nervous system disorders	11 (58)	7 (37)	4 (29)
Headache	6 (32)	4 (21)	2 (14)
Respiratory, thoracic, and mediastinal disorders	10 (53)	7 (37)	4 (29)
Vascular disorders	8 (42)	3 (16)	4 (29)
Hepatobiliary disorders	8 (42)	6 (32)	3 (21)
Hepatic function abnormal	8 (42)	3 (16)	0
Blood and lymphatic system disorders	8 (42)	6 (32)	2 (14)
Thrombocytopenia	5 (26)	1 (5)	0
Ear and labyrinth disorders	5 (26)	1 (5)	2 (14)
Investigations^b^	7 (37)	6 (32)	2 (14)
Injury, poisoning, and procedural complications	7 (37)	5 (26)	1 (7)
Psychiatric disorders	5 (26)	2 (11)	1 (7)
Renal and urinary disorders	5 (26)	3 (16)	1 (7)

Data presented as *n* (%).

a Reported by ≥ 25% of patients during 0 to 2, > 2 to 4, or > 4 years of treatment.

b Refers to the Medical Dictionary for Regulatory Activities system-organ class category for the names and qualitative results (eg, increased, decreased, normal, abnormal, present, absent, positive, negative) of investigations, including clinical laboratory test concepts (eg, biopsies), radiologic test concepts, physical examination parameters, and physiologic test concepts (eg, a pulmonary function test).

Also for the combined phase 1 and extension studies, 5 (26%) patients had SAEs, including 3 (16%) patients with SAEs during the extension study. Two of these SAEs (syncope and dyspnea) were considered unrelated to siltuximab, and 1 SAE (polycythemia) was considered at least possibly attributed to siltuximab. Twenty days after the third dose of siltuximab in the extension study (and also following 31 doses in the phase 1 study), a male patient developed grade-3 secondary polycythemia (hemoglobin of 18.8 g/dL). This SAE led to hospitalization, resolved with hydration and anti-coagulation treatment without complications, and did not recur despite continued siltuximab treatment. Following a 3-week dose delay, this patient continued siltuximab and has received 25 cycles (total of 56 doses including phase 1 exposure) as of the cut-off date for this safety analysis.

Nine (47%) patients had a dose delay due to an AE, 4 of which occurred during the extension study and 1 of which was due to an AE possibly attributed to siltuximab; no patient discontinued siltuximab due to an AE.

Infections, hyperlipidemia, neutropenia, thrombocytopenia, gastrointestinal perforations, infusion-related reactions, and liver function abnormalities were further analyzed as AEs of interest in this long-term safety analysis (Table 3). Most infections were low-grade. Common infections, irrespective of grade, for the extension study were upper respiratory tract infection (63%), sinusitis (11%), nasopharyngitis (11%), and pneumonia (11%). Two (11%) of the extension-study patients reported 1 or more serious infections during the phase 1 study (abscess limb, cellulitis, device-related infection, peritoneal infection, pyelonephritis, vulval abscess, staphylococcal wound infection); no serious infections were reported during the extension study. No unusual viral infections or reactivations were observed; 7 (37%) patients had—and are currently receiving—prophylactic acyclovir or valacyclovir; 5 (26%) patients were receiving antivirals at baseline, prior to any siltuximab treatment, and 6 (32%) were receiving antiviral prophylaxis during the extension study. Similarly, no opportunistic fungal infections were seen. Across the combined phase 1 and extension studies, the overall incidence of grade-≥3 serious infections was 0.02 per patient-year.

**Table 3:** Frequently reported^a^ adverse events by system-organ class and study period

	Study period^b^					
	Phase 1 (*n* = 19)		Extension (*n* = 19)		Combined (*n* = 19)	
	All Grades	Grade ≥ 3	All Grades	Grade ≥ 3	All Grades	Grade ≥ 3
Patients with adverse events	19 (100)	10 (53)	19 (100)	8 (42)	19 (100)	12 (63)
Infections and infestations	17 (90)	5 (26)	17 (90)	0	19 (100)	5 (26)
Upper respiratory tract infection	11 (58)	0	12 (63)	0	17 (90)	0
Sinusitis	7 (37)	0	2 (11)	0	7 (37)	0
Nasopharyngitis	4 (21)	0	2 (11)	0	6 (32)	0
Urinary tract infection	5 (26)	0	1 (5)	0	5 (26)	0
Ear infection	3 (16)	0	1 (5)	0	4 (21)	0
Gastrointestinal disorders	16 (84)	6 (32)	12 (63)	0	17 (90)	6 (32)
Nausea	12 (63)	2 (11)	3 (16)	0	12 (63)	2 (11)
Vomiting	11 (58)	1 (5)	1 (5)	0	11 (58)	1 (5)
Diarrhea	9 (47)	1 (5)	6 (32)	0	10 (53)	1 (5)
Constipation	5 (26)	0	2 (11)	0	6 (32)	0
Abdominal pain	3 (16)	1 (5)	2 (11)	0	5 (26)	1 (5)
Dyspepsia	3 (16)	0	2 (11)	0	4 (21)	0
Metabolism and nutrition disorders	17 (90)	0	4 (21)	0	17 (90)	0
Hypercholesterolemia	9 (47)	0	0	0	9 (47)	0
Hypertriglyceridemia	8 (42)	0	1 (5)	0	8 (42)	0
Hyperuricemia	7 (37)	0	0	0	7 (37)	0
Hypokalemia	4 (21)	0	1 (5)	0	5 (26)	0
Hypomagnesaemia	4 (21)	0	0	0	4 (21)	0
Musculoskeletal and connective tissue disorders	15 (79)	1 (5)	9 (47)	0	16 (84)	1 (5)
Pain in extremity	7 (37)	0	4 (21)	0	8 (42)	0
Arthralgia	6 (32)	0	4 (21)	0	7 (37)	0
Back pain	5 (26)	0	3 (16)	0	7 (37)	0
Muscle spasms	4 (21)	0	1 (5)	0	5 (26)	0
General disorders and administration site conditions	9 (47)	3 (16)	8 (42)	1 (5)	13 (68)	4 (21)
Fatigue	2 (11)	1 (5)	4 (21)	1 (5)	5 (26)	2 (11)
Peripheral Edema	4 (21)	0	1 (5)	0	5 (26)	0
Nervous system disorders	11 (58)	2 (11)	7 (37)	1 (5)	12 (63)	3 (16)
Headache	6 (32)	1 (5)	3 (16)	0	8 (42)	1 (5)
Dizziness	5 (26)	0	0	0	5 (26)	0
Hypoesthesia	4 (21)	1 (5)	0	0	4 (21)	1 (5)
Respiratory, thoracic, and mediastinal disorders	12 (63)	2 (11)	6 (32)	1 (5)	12 (63)	3 (16)
Cough	5 (26)	0	0	0	5 (26)	0
Oropharyngeal pain	5 (26)	0	0	0	5 (26)	0
Skin and subcutaneous tissue disorders	9 (47)	1 (5)	7 (37)	0	12 (63)	1 (5)
Rash	6 (32)	1 (5)	3 (16)	0	8 (42)	1 (5)
Vascular disorders	10 (53)	3 (16)	3 (16)	0	12 (63)	3 (16)
Hypertension	4 (21)	3 (16)	1 (5)	0	5 (26)	3 (16)
Hot flush	2 (11)	0	2 (11)	0	4 (21)	0
Investigations^c^	9 (47)	1 (5)	5 (26)	1 (5)	11 (58)	1 (5)
Blood and lymphatic system disorders	9 (47)	1 (5)	4 (21)	3 (16)	10 (53)	4 (21)
Thrombocytopenia	5 (26)	0	0	0	5 (26)	0
Hepatobiliary disorders	8 (42)	1 (5)	3 (16)	0	9 (47)	1 (5)
Hepatic function abnormal	8 (42)	1 (5)	0	0	8 (42)	1 (5)
Hyperbilirubinemia	3 (16)	0	3 (16)	0	5 (26)	0
Renal and urinary disorders	8 (42)	1 (5)	1 (5)	0	9 (47)	1 (5)
Renal impairment	4 (21)	0	0	0	4 (21)	0
Injury, poisoning, and procedural complications	7 (37)	1 (5)	4 (21)	0	8 (42)	1 (5)
Reproductive system and breast disorders	5 (26)	0	3 (16)	0	8 (42)	0
Psychiatric disorders	6 (32)	1 (5)	1 (5)	0	7 (37)	1 (5)
Anxiety	5 (26)	1 (5)	0	0	5 (26)	1 (5)
Depression	4 (21)	0	0	0	4 (21)	0
Ear and labyrinth disorders	5 (26)	2 (11)	2 (11)	0	6 (32)	2 (11)
Eye disorders	3 (16)	0	5 (26)	1 (5)	6 (32)	1 (5)

Data presented as *n* (%).

a Reported by ≥ 20% of patients in either phase 1, extension study, or the combined phase 1 and extension studies.

b Refers to study period (CNTO328T03 [phase 1] vs. CNTO328MCD2002 [extension study]) during which the AE(s) occurred; “Phase 1” refers to patients enrolled in the phase 1 study who continued on to the extension study.

c Refers to the Medical Dictionary for Regulatory Activities system-organ class category for the names and qualitative results (eg, increased, decreased, normal, abnormal, present, absent, positive, negative) of investigations, including clinical laboratory test concepts (eg, biopsies), radiologic test concepts, physical examination parameters, and physiologic test concepts (eg, a pulmonary function test).

**Figure 2:**
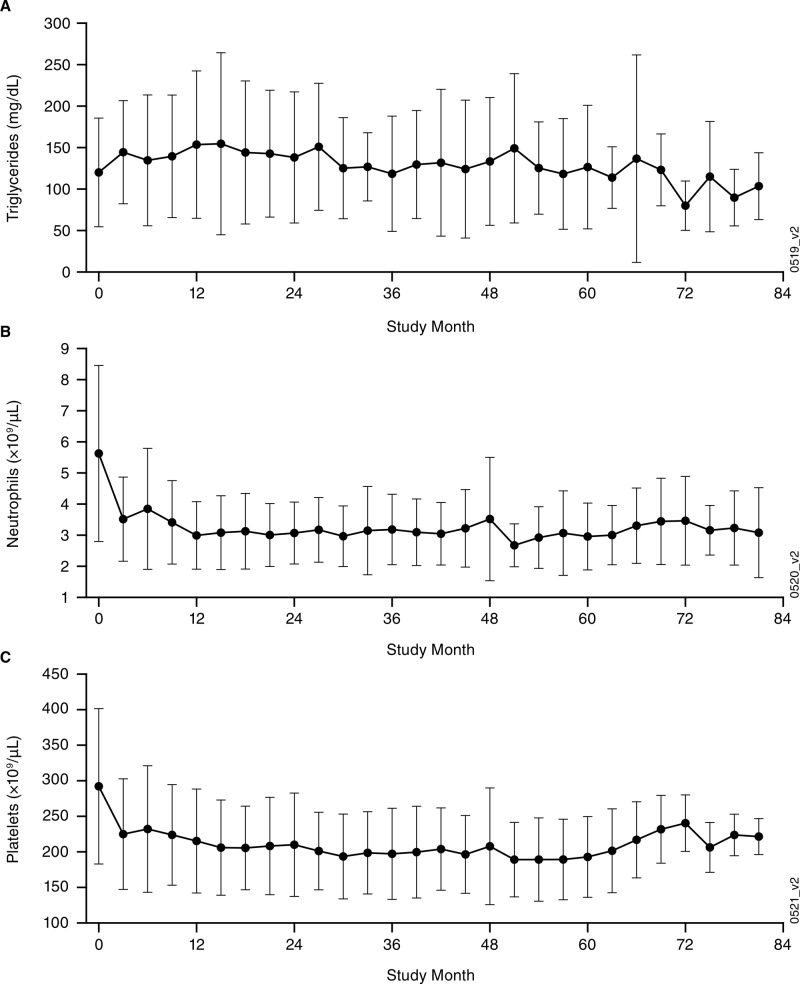
Clinical laboratory findings (mean ± standard deviation [SD]) over time for the combined phase 1 and extension studies **A.** triglycerides, **B.** neutrophils, and **C.** platelets. Of the 19 extension study patients, 6 (32%) had been receiving lipid-modifying therapy prior to the phase 1 study and 9 (47%) received such therapy during the extension study.

Although hypercholesterolemia (total cholesterol; 47%) and hypertriglyceridemia (42%) were common among these 19 patients during the phase 1 study, these elevations were low-grade (9 grade-1 hypercholesterolemia; 5 grade-1 and 3 grade-2 hypertriglyceridemia); 1 patient had hypertriglyceridemia during the extension study. The overall incidence of all-grade hypertriglyceridemia was 0.13 per patient-year. Of the 19 patients, 6 (32%) had been receiving lipid-modifying therapy prior to the phase 1 study and 9 (47%) received such therapy during the extension study. Two patients reported neutropenia, including 1 grade-3 event, during the extension study; the overall incidence of grade-≥3 neutropenia was 0.01 per patient-year. Thrombocytopenia (26%) was reported only during the phase 1 study and with no grade-≥3 events. Because hypertriglyceridemia has previously been observed in patients who received siltuximab [5, 6] or other IL-6-inhibiting agents, such as tocilizumab [10, 11], and because siltuximab's mechanism of action involving IL-6 blockade may decrease platelet and neutrophil counts in patients, mean values over time for triglycerides, neutrophils, and platelets were recorded and are shown in Figure 2A–2C. Hepatic function abnormalities were reported in 8 (42%) patients, including 3 grade-1, 4 grade-2, and 1 grade-3 events during the phase 1 study, while none were reported during the extension study. Of the 8 patients with hepatic function abnormalities, 3 had alanine aminotransferase elevations and 4 had aspartate aminotransferase elevations prior to receiving siltuximab, while none had abnormal bilirubin prior to receiving siltuximab. Three patients had grade-2 abnormal total bilirubin during the phase 1 study. No patients discontinued treatment with siltuximab or had dose delays due to hepatic abnormalities.

### Efficacy

At the time of extension study start, 1 patient had CR, 11 had PR, and 7 had stable disease (SD) based on independent review of radiographic images obtained during the phase 1 study. During the extension study, investigator assessment of disease control was documented. All 19 patients continued to have sustained disease control, including 8 patients whose treatment interval was lengthened from q3wks to q6wks due to established prolonged CR. The one additional patient who started in the extension study on a q6wks dosing interval showed an increase of clinical symptoms and was switched back to q3wks dosing after 4 infusions, upon which the symptoms resolved. The median duration of disease control from the start of the extension study was 1.5 (range 1.4, 1.6) years. As such, the overall survival rate in these 19 patients following a median of 5.1 years of follow-up in the combined phase 1 and extension studies is 100%.

## DISCUSSION

We have previously reported on a phase 1 study [5] comprising 37 patients with Castleman disease treated with the anti-IL-6 monoclonal antibody siltuximab, which directly neutralizes IL-6, a key cytokine in the pathogenesis of MCD. Nineteen patients experienced significant clinical benefit from siltuximab treatment and transitioned to an extension study in order to continue receiving therapy. Disease control was maintained in all patients during the extension study, and with a median follow-up of 5.1 (range 3.4, 7.2) years from the onset of siltuximab, the overall survival rate of these 19 patients was 100%, with nearly three quarters of patients receiving treatment for longer than 4 years. The efficacy of siltuximab in MCD has recently been confirmed in an international randomized, double-blind, placebo-controlled study [7], which has led to the first regulatory approval of siltuximab for MCD in the US [8] and European Union [9]. Both in the phase 1 [5] and subsequent randomized study [7], in addition to radiologic response and symptom control, significant improvement in inflammatory markers and normalization of hemoglobin and albumin were also noted.

The present cohort of patients with MCD received a median of 81 (range 49–129) siltuximab doses. Overall, siltuximab was well-tolerated, and none of the patients discontinued therapy. During the study period of >5 years, the most common AEs of any grade were upper respiratory tract infection, nausea, vomiting, diarrhea, and hypercholesterolemia (total cholesterol). Adverse events that occurred during the extension study and were considered at least possibly attributed to siltuximab included pneumonia, tooth abscess, constipation, leukopenia, lymphopenia, increased hemoglobin, hypertriglyceridemia, hyperbilirubinemia, chronic otitis media, and upper respiratory tract infection. Incidence of SAEs was low; during the extension study, 1 patient also developed a grade-3 SAE, polycythemia, which was considered treatment-related. Over time, the blood counts remained stable, and no unusual opportunistic infections were observed. The overall incidence of grade-≥3 neutropenia was 0.01/patient-year, and the incidence of grade-≥3 serious infections was 0.02/patient-year. Low-grade elevation of total cholesterol and triglycerides was common—47% and 42%, respectively—but all were manageable with lipid modified agents; 6 (32%) patients received lipid-modifying agents prior to siltuximab treatment and 9 (47%) received them during the extension study. A BMI > 30 was recorded in 7 of the 19 patients prior to siltuximab treatment and in 8 of 19 patients at the start of the extension study. The incidences of AEs occurring ≤2, >2–4, and >4 years after initiation of siltuximab therapy were similar, indicating no cumulative toxicity. No infusion reactions or development of antibodies against siltuximab were noted during this extension study. In total, 9 patients were dosed at q6wks intervals from the start of the study or switched during the study. Eight of them maintained q6wks dosing throughout the study. One patient had to return to a q3wks treatment interval for symptom control that was maintained with continued dosing at q3wks. Per protocol, patients were only allowed to receive treatment either q3wks or q6wks.

Of note, these findings are limited in that they present on an open-label, single-arm extension study offered to patients who were responding to siltuximab treatment. Nonetheless, long-term siltuximab administration was associated with effective long-term disease control with a favorable toxicity profile including when administered over years and is an important addition to the therapeutic armamentarium for MCD.

## MATERIALS AND METHODS

### Ethics statement

Investigation has been conducted in accordance with the ethical standards and according to the Declaration of Helsinki and to national and international guidelines and has been approved by the investigators’ institutional review boards. All patients provided written informed consent.

### Patients

Patients with MCD previously enrolled in the open-label phase 1 siltuximab study were 18 years or older, HIV-negative and HHV-8-negative, and presented with active symptomatic MCD. Patients were eligible for the extension study if: 1) their disease did not progress while receiving siltuximab, 2) they had no clinically significant toxicity of grade 2 or higher than the baseline value from the phase 1 study, and 3) they received the last siltuximab administration within 6 ± 2 weeks before the first study extension dose was administered.

### Study design

This is an ongoing, open-label, multicenter, single-arm, phase 2 extension study (NCT01400503) to assess the safety of long-term treatment with siltuximab in MCD patients who were previously treated in a siltuximab phase 1 study (NCT00412321). The preplanned safety analysis reported here was conducted 21 months after the start of patient enrollment in the extension study and includes 19 patients from the preceding phase 1 siltuximab study.

Patients received siltuximab as a 1-hour intravenous infusion at 11 mg/kg every 3 weeks (q3wks). The treatment interval could be lengthened to 6 weeks (q6wks) at the investigator's discretion for patients with documented radiographic response (confirmed partial [PR] or complete [CR] response) for >6 months. Dose delays of up to 3 weeks were permitted for management and/or resolution of AEs. All patients were treated with study agent until they progressed, withdrew consent, or experienced unacceptable toxicity.

### Study evaluations

Adverse events were graded according to the National Cancer Institute Common Terminology Criteria for Adverse Events, version 4.0. Physical examinations and clinical hematology and chemistry tests were performed prior to the cycle 1 dose and every 3 cycles thereafter for patients receiving the q3wks regimen or every other dosing visit for patients receiving the q6wks regimen. Lipid panel analyses were done prior to cycle 1 dose and every 6 months thereafter. Investigators assessed disease control during screening; at cycles 4, 7, and 10; every 6 months thereafter; and within 4 weeks following the time of siltuximab discontinuation. Laboratory assessments for erythrocyte sedimentation rate (ESR), CRP, and fibrinogen were performed at these same timepoints. For those who discontinued treatment, survival status and subsequent MCD therapies were to be collected until the patient was lost to follow-up or withdrew consent for the study, until 50% of patients died, or until the end of the study—whichever occurred first. Survival status will be collected twice a year for patients remaining on study treatment after the 5-year data cutoff. For this interim analysis, duration of follow-up and overall survival—defined as time between the first study siltuximab administration and death—are reported. Duration of disease control is reported only for the duration of the MCD2002 study.

### Statistical analysis

Descriptive statistics were used to summarize data. No formal hypothesis testing was performed. The sample size of this extension study was determined by the eligibility of patients transitioning from the phase 1 to the phase 2 study and not dictated by statistical calculation. Demographics and disease characteristics were reported from the phase 1 study [5]. Exposure and safety data from the 19 patients who were enrolled in the phase 1 study and in the extension study were combined for analysis. Due to the long treatment duration, safety analyses included both cumulative incidence for all AEs and incidence per patient-years of exposure for AEs of clinical interest. Efficacy was assessed using central imaging data during the phase 1 study and using the investigator's assessment of disease control (specifically, proportions of previously-responding patients and siltuximab-naïve patients who maintained disease control) and duration of disease control during the phase 2 extension study.
